# It’s All in Your Head: A Case Report of Persistent Postural-Perceptual Dizziness and Chronic Anxiety Treated Simultaneously With Osteopathic Cranial Manipulative Medicine

**DOI:** 10.7759/cureus.110597

**Published:** 2026-06-10

**Authors:** Abraham E Libman, Mikhail I Volokitin, David Lee, Katelyn Wang, Daniella Maydan, Tzipora Benyaminov, Laura N Tarbay, Katherine M Petrov

**Affiliations:** 1 Osteopathic Manipulative Medicine, Touro College of Osteopathic Medicine, New York, USA

**Keywords:** cranial somatic dysfunction, diagnostic overshadowing, osteopathic structural exam, persistent postural-perceptual dizziness (pppd), superior sphenoid strain

## Abstract

Persistent postural-perceptual dizziness is a chronic functional vestibular disorder characterized by persistent dizziness, imbalance, motion sensitivity, and significant psychological distress, frequently including anxiety. Symptoms often persist despite normal imaging and multidisciplinary evaluation, contributing to diagnostic uncertainty and misattribution to psychiatric causes. Osteopathic cranial manipulative medicine is not routinely incorporated into the management of persistent postural-perceptual dizziness despite osteopathic principles, emphasizing the interrelationship between cranial structure, autonomic regulation, vestibular function, and psychological well-being.

A 30-year-old female patient with a longstanding history of anxiety, depression, and post-traumatic stress disorder presented with a greater than two-year history of persistent postural-perceptual dizziness, recurrent falls, and imbalance that began following a motor vehicle accident. Her psychiatric symptoms had been refractory to psychotherapy and pharmacologic treatment, while her imbalance remained refractory to physical therapy and neurologic evaluation. The chronic persistence of vestibular symptoms coincided with worsening psychological distress and functional decline. Magnetic resonance imaging demonstrated a suprasellar pituitary lesion without a definitive explanation for her symptoms. Osteopathic structural examination identified decreased cranial rhythmic impulse amplitude, increased dural tension, and superior sphenoid strain at the sphenobasilar synchondrosis (SBS). Osteopathic manipulative treatment was performed, including osteopathic cranial manipulative medicine techniques directed at reducing dural tension and restoring balanced motion at the cranial base. Two treatment sessions were performed one week apart.

Following the first treatment session, the patient reported mild improvement in balance and substantial reduction in anxiety symptoms. Following the second session, she reported further improvement in balance, anxiety, and ambulatory confidence compared with pretreatment baseline. Generalized Anxiety Disorder-7 scores improved to below the screening threshold for generalized anxiety disorder, while Patient Health Questionnaire-9 score improved from 11, consistent with moderate depression, to 5, consistent with mild depressive symptoms. The patient additionally reported fewer falls, improved confidence with ambulation, and immediate subjective relief following treatment.

This case suggests a potential role of osteopathic cranial somatic dysfunction in persistent postural-perceptual dizziness and associated psychological distress refractory to conventional management. Identification and treatment of superior sphenoid strain were temporally associated with clinically meaningful improvements in vestibular symptoms, anxiety, depressive symptoms, and functional capacity. These findings support further investigation into osteopathic cranial manipulative medicine as a potential adjunctive treatment approach for persistent postural-perceptual dizziness and related neuropsychiatric symptomatology. This case also highlights the importance of comprehensive, multidisciplinary evaluation in patients whose physical symptoms may be prematurely attributed solely to psychiatric diagnoses.

## Introduction

Background

Diagnostic overshadowing refers to the misattribution of new or worsening physical symptoms to psychiatric causes instead of conducting a thorough medical evaluation, resulting in missed or delayed diagnoses, reduced diagnostic testing, and inadequate treatment. From patient perspective, this well-documented phenomenon is both common and harmful. Surveys of individuals with psychiatric conditions demonstrate that physical complaints are frequently dismissed as manifestations of anxiety or depression, with subsequent identification of missed infections, neurological disorders, and organ disease [1]. Patients report being told that symptoms are “just anxiety” or “all in their head,” experiences which can cause loss of trust in clinicians and reluctance to seek further medical care. These experiences are often internalized, leading patients to question their own symptoms, minimize concerns, or withhold psychiatric history in an attempt to avoid bias [2].

Clinician-driven factors play a crucial role in perpetuating diagnostic overshadowing. Systematic reviews of healthcare encounters reveal that negative stereotypes toward patients with mental illness, such as assumptions of unreliability or symptom exaggeration, significantly influence diagnostic decision-making [3]. Interviews with emergency physicians and nurses show that many clinicians acknowledge prematurely writing off physical complaints to psychiatric causes and “closing off” diagnostic pathways early, even though they recognized that this practice could result in delayed treatment and worse outcomes [3]. Implicit bias further compounds this issue, with studies revealing that healthcare professionals often perform less thorough diagnostic evaluations on patients with histories of psychiatric disorders and that these biases are reinforced by peer attitudes and institutional culture [4].

At a systemic level, diagnostic overshadowing contributes to broader health disparities among individuals with mental illness. Reviews of healthcare outcomes across multiple medical conditions show that 78% of studies report higher rates of missed or delayed diagnoses in patients with psychiatric histories compared to the general population, including later-stage cancer diagnoses, undertreated pain, and misdiagnosed neurological disease [5]. These findings suggest that diagnostic inequities are driven not solely by clinical complexity but by structural stigma embedded within healthcare systems. Together, this body of evidence underscores the importance of diagnostic approaches that acknowledge psychiatric history without allowing it to eclipse comprehensive evaluation of physical contributors to illness.

Persistent postural-perceptual dizziness and osteopathic considerations

Persistent postural-perceptual dizziness (PPPD) has emerged as a contemporary diagnostic framework for chronic functional dizziness syndromes and is characterized by persistent non-spinning dizziness, postural instability, motion sensitivity, and visual dependence lasting at least three months. Symptoms typically occur daily and intensify with upright posture, active or passive movement, and exposure to visually complex environments. PPPD is increasingly understood as a disorder involving altered sensory integration, autonomic dysregulation, and anxiety-related mechanisms, and often emerges following a vestibular, neurological, or psychological precipitant. It is commonly associated with anxiety, depression, and trauma-related disorders. Diagnosis remains challenging, as standard neuroimaging and vestibular testing are usually normal, and treatment responses to conventional interventions such as vestibular rehabilitation, psychotherapy, and pharmacologic management are variable. Because PPPD frequently overlaps with psychiatric, neurologic, vestibular, autonomic, and structural symptoms, evaluation often requires an integrative and multidisciplinary clinical approach, particularly in patients with coexisting endocrine or neuroanatomical abnormalities [2-6].

Osteopathic cranial manipulative medicine (OCMM) is not routinely incorporated into the evaluation or management of PPPD. However, osteopathic medicine emphasizes the interrelationship between structure and function, particularly at the cranial base, where biomechanical relationships influence vestibular pathways and autonomic imbalance [6]. The superior sphenoid plays an integral role in cranial mechanics, articulating with multiple bones of the cranial base and transmitting forces that may affect neural and vascular structures related to balance and perception. Dysfunction at this level may not be detected through conventional imaging; however, it may be identified through osteopathic cranial examination. Because PPPD frequently overlaps with anxiety and trauma-related symptoms, standardized screening tools such as the Patient Health Questionnaire-9 (PHQ-9) and Generalized Anxiety Disorder-7 (GAD-7) can help quantify psychological distress over time and reduce reliance on subjective attribution of symptoms to psychiatric history alone. Osteopathic medicine emphasizes a whole-person approach, including the role of somatic dysfunction and autonomic balance in contributing to physical symptoms and overall functional impairment [6].

Given the overlap between vestibular, psychiatric, autonomic, and structural symptoms in PPPD, osteopathic structural evaluation may provide an additional framework for assessing potential somatic contributors in select patients. The objective of this case report is to describe a medically complex patient with chronic dizziness, psychiatric comorbidity, and sellar pathology whose symptoms improved temporally following OCMM, while also highlighting the diagnostic challenges involved in differentiating functional and organic contributors to persistent vestibular symptoms.

## Case presentation

A 30-year-old female patient presented to an osteopathic physician with a more than two-year history of persistent balance disturbance, chronic dizziness, and progressive functional decline. Her chief complaint consisted of frequent dizziness occurring multiple times daily, particularly with positional changes such as rising from seated or supine positions. She also reported an intermittent sensation of leaning forward and toward the left, as well as episodes of unpredicted unsteadiness throughout the day. These symptoms significantly impaired her ability to ambulate independently and resulted in recurrent falls several times per week. Ambulation with a cane reduced fall frequency but did not eliminate episodes of instability.

Medical and psychiatric history

The patient’s past medical history was notable for PPPD. She had been involved in a motor vehicle accident two years prior to her visit, coinciding with the onset of her balance issues. Although the temporal relationship between the accident and symptom onset raised consideration of post-traumatic vestibular or neurologic sequelae, the persistence of chronic non-spinning dizziness, postural instability, motion sensitivity, and visually exacerbated symptoms in the setting of largely unrevealing neurologic and imaging evaluations contributed to the working clinical diagnosis of PPPD. Subsequent imaging studies did not reveal acute traumatic injury. Her PPPD was diagnosed clinically based on chronic dizziness, postural instability, and balance disturbance; however, formal vestibular testing, including videonystagmography/electronystagmography, vestibular evoked myogenic potentials, video head impulse testing, and computerized posturography, was not documented in the available records. The absence of comprehensive vestibular testing limits diagnostic certainty and the ability to fully exclude alternative vestibular or neurologic etiologies. Despite this clinical diagnosis and implementation of recommended management strategies, including psychotherapy and physical therapy, her symptoms remained refractory.

Her medical history was also significant for hypothyroidism, managed with Levothyroxine sodium (T4) and Levothyroxine + liothyronine (T4 + T3 combination), and a benign pituitary lesion was identified on neuroimaging during prior evaluations. Surgical intervention was not recommended given the absence of clear endocrine or neurological indications. It was noted that the lesion could potentially influence pituitary hormone regulation, although no definitive causal relationship to her presenting symptoms was established. The pituitary gland is located within the sella turcica of the sphenoid bone, a region closely associated with surrounding neurovascular structures.

Her psychiatric history also included a long-standing history of anxiety, depression, and post-traumatic stress disorder (PTSD) related to adverse childhood experiences. These psychiatric conditions had been present for over a decade prior to the onset of her balance symptoms. She had been evaluated and treated by a psychiatrist and had tried multiple anxiolytic and antidepressant medications, including venlafaxine and quetiapine. Despite psychiatric management, her dizziness and imbalance persisted.

Family history included lung and breast cancers and facioscapulohumeral muscular dystrophy. The patient’s electromyography test and creatine phosphokinase test came back normal, while two prior genetic evaluations were nondiagnostic. Her relevant laboratory and diagnostic findings are summarized in Table 1.

**Table 1:** Laboratory and diagnostic evaluation (endocrine and neuromuscular) Note: Thyroid function results reflect known hypothyroidism managed with hormone replacement therapy. TSH, thyroid-stimulating hormone; LH, luteinizing hormone; FSH, follicle-stimulating hormone; IGF-1, insulin-like growth factor-1; CPK, creatine phosphokinase; EMG, electromyography

Test	Result	Reference Range	Timing	Interpretation
TSH, uIU/mL	0.01	0.55–4.78	Follow-up	Low (on thyroid replacement)
Free T4, pmol/L	17.2	11.5–22.7	Follow-up	Normal
Free T3, pmol/L	5.42	3.5–6.5	Follow-up	Normal
Prolactin, ng/mL	9.9	2.8–29.2	Follow-up	Normal
LH, IU/L	15.1	Varies by cycle	Follow-up	Within the expected range
FSH, IU/L	9.32	Varies by cycle	Follow-up	Within the expected range
Cortisol, µg/dL	7.38	4.3–22.4	Follow-up	Normal
Growth hormone, ng/mL	0.2	<8	Follow-up	Normal
IGF-1, ng/mL	166	Age-dependent	Follow-up	Normal
CPK, U/L	Normal	10–120	Prior evaluation	Normal
EMG	Normal	—	Prior evaluation	No neuromuscular abnormality

These findings did not suggest an alternative metabolic, endocrine, or neuromuscular etiology for the patient’s symptoms.

Prior evaluations and management

Over the course of two years, the patient underwent multidisciplinary evaluation involving primary care, neurology, psychiatry, endocrinology, neurosurgical consultation, and physical therapy for persistent dizziness, imbalance, psychiatric symptoms, and pituitary findings. A year prior to osteopathic structural examination, neuroimaging revealed a pituitary lesion that was considered radiographically benign and not definitively explanatory of the patient’s full clinical presentation. No surgical intervention or medical treatment was recommended, as removal would require invasive neurosurgery, and there was no clear evidence that the lesion fully accounted for her symptoms. At the time of evaluation, the pituitary lesion was not felt by the treating specialists to fully account for the severity and chronicity of the patient’s vestibular and functional symptoms. Although the lesion remained clinically relevant and required continued monitoring, the available endocrine, neurologic, and imaging evaluations did not establish a definitive causal explanation for the full severity of the patient’s vestibular and functional symptoms.

Magnetic resonance imaging (MRI) demonstrated mild suprasellar involvement of the pituitary gland with a central stalk that remained midline but was compressed anteriorly. Minimal pressure/contact on the optic chiasm was newly identified compared with prior imaging. The pituitary process was described in the radiology report as demonstrating slight interval enlargement between examinations. The radiology differential diagnosis included a Rathke cleft cyst versus a pituitary adenoma containing proteinaceous material or blood breakdown products, and neurosurgical evaluation was recommended for clinical correlation. The patient’s clinical course is summarized in Figure 1, and relevant MRI findings from the patient’s medical records are summarized below and shown in Figures 2-4.

**Figure 1:**
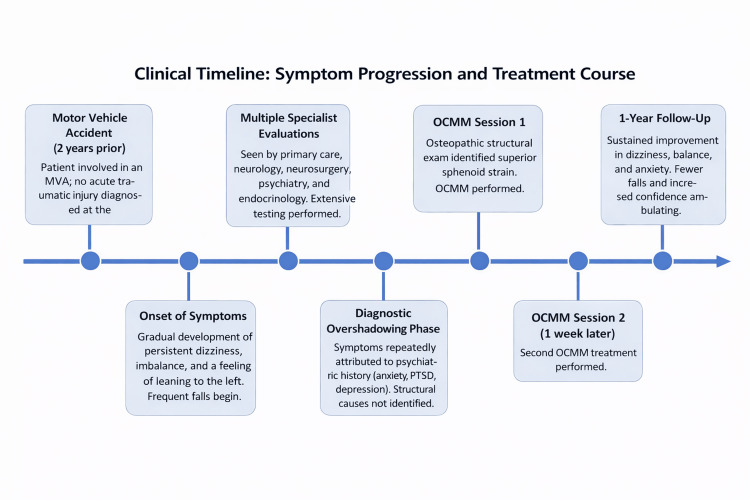
Clinical timeline of symptom progression, diagnostic course, and osteopathic treatment interventions. Figure created using Microsoft PowerPoint. MVA, motor vehicle accident; OCMM, osteopathic cranial manipulative medicine; PTSD, post-traumatic stress disorder

**Figure 2:**
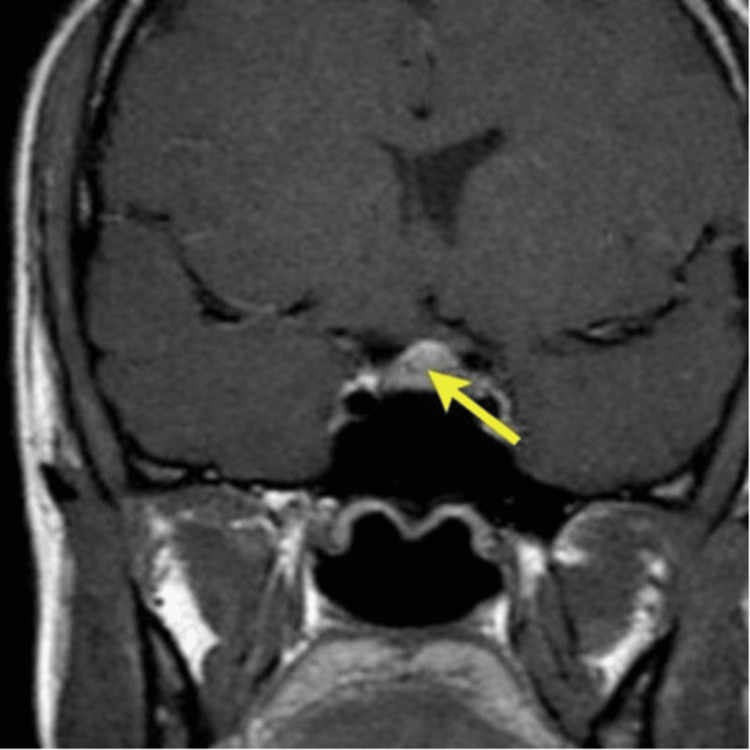
Coronal T1-weighted MRI of the sellar region demonstrating a pituitary lesion with mild suprasellar involvement and new minimal interval contact/mass effect on the optic chiasm (arrow), as documented in the patient’s medical records.

Additional sagittal and axial views further demonstrated the sellar/suprasellar relationship described in the radiology report (Figures 3, 4).

**Figure 3:**
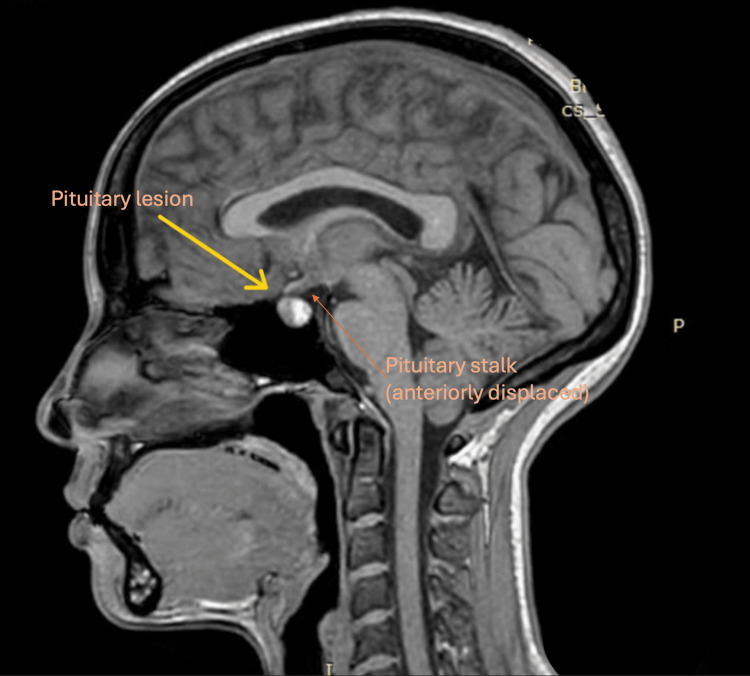
Sagittal MRI of the sella demonstrating a pituitary lesion with mild suprasellar extension. Per the radiology report, the lesion demonstrated slight interval growth with anterior displacement of the pituitary stalk and minimal new interval contact/pressure on the optic chiasm.

**Figure 4:**
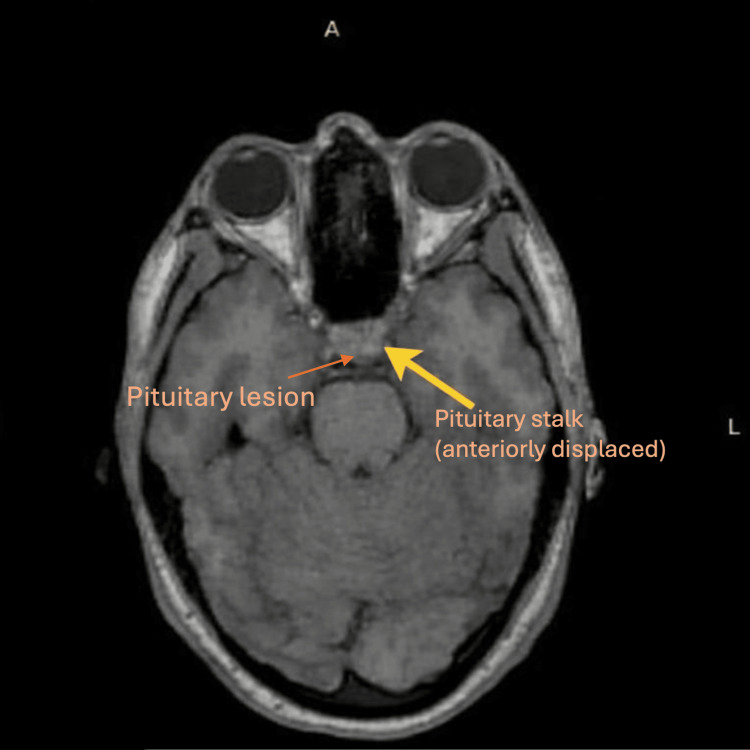
Axial T1-weighted MRI of the brain centered on the sellar/suprasellar region demonstrating a pituitary lesion (arrow) and corresponding to the pituitary process referenced in the radiology report.

Despite extensive evaluation, there was no structural or neurological cause identified to explain the patient’s persistent dizziness. Her symptoms were repeatedly attributed to psychiatric etiologies, including anxiety and PTSD. Management strategies primarily focused on psychiatric treatment including psychotherapy, physical therapy, and pharmacologic interventions aimed at improving balance and strength. Psychotherapy and psychiatric management had been ongoing for several years prior to presentation, while physical therapy focused on balance rehabilitation was pursued without substantial improvement in dizziness, instability, or fall frequency. These strategies were proven ineffective as the patient experienced minimal to no improvement in symptoms.

As her condition progressed, the patient reported recurrent falls occurring several times per week, resulting in secondary musculoskeletal injuries and increasing functional impairment. She became unfit to drive, experienced disrupted sleep, missed work obligations, and developed a significant fear of ambulation. She required a cane for ambulation and later progressed to the use of a mobility scooter to reduce her risk of falling. The cumulative impact of these limitations contributed to her social withdrawal and further exacerbated her anxiety and depressive symptoms.

Osteopathic evaluation

After persistent symptoms refractory to conventional management, the patient underwent a comprehensive osteopathic evaluation performed by an osteopathic physician with prior postgraduate training and clinical experience in OCMM. The evaluation included a full osteopathic structural examination assessing posture, gait mechanics, spinal motion, extremity alignment, and cranial mechanics, as well as palpation of the cranial rhythmic impulse (CRI).

The osteopathic structural examination identified several somatic dysfunctions that can be potentially explained by the patient’s history of recurrent falls and postural instability. These included a functional type I thoracic curve from T4 to T9 (neutral, side bent left, rotated right), repeated ankle inversion injuries, and a functional short right lower extremity measuring approximately 1.5 cm shorter than the left. These somatic dysfunctions were addressed with osteopathic manipulative treatment, including muscle energy and high-velocity, low-amplitude techniques. These treatments resulted in the resolution of localized musculoskeletal pain and improved structural symmetry.

In addition to the structural findings, the cranial examination revealed an abnormal CRI characterized by decreased amplitude and altered quality, along with increased dural tension and asymmetry at the cranial base. Palpatory findings were consistent with a superior sphenoid strain pattern, identified by restricted motion at the SBS and a vertical shearing motion appreciated during CRI assessment. These findings were interpreted within the osteopathic cranial framework as potentially relevant to the patient’s chronic dizziness, postural instability, and autonomic symptoms.

Osteopathic cranial manipulative medicine

Based on the osteopathic evaluation, a course of OCMM was initiated to treat the identified superior sphenoid strain. For this patient, OCMM was directed at restoring normal motion at the SBS and reducing associated dural tension. Treatment techniques included SBS decompression, in which general axial traction was applied to promote improved separation and reciprocal motion between the sphenoid and occiput. Balanced membranous tension techniques were also employed to address strain within the dural membranes by applying reciprocal tensile forces to regions of restriction to normalize dural tension and support physiologic cranial motion. Compression of the fourth ventricle was utilized as an indirect cranial technique to support parasympathetic tone and normalize the CRI, consistent with foundational osteopathic teaching. All techniques were administered using gentle manual force over several treatment cycles and were guided by continuous palpatory reassessment of cranial motion and CRI quality throughout the treatment sessions.

Outcome

Two OCMM treatment sessions were performed one week apart. Following the first session, the patient self-reported 10% improvement in balance and 50% reduction in anxiety. After the second session, she reported 25% improvement in balance and 75% reduction in anxiety since before initial treatment. Follow-up psychiatric screening demonstrated normalization of the GAD-7 score and a reduction in the PHQ-9 score from 11 (consistent with moderate depression) to 5 (consistent with mild depressive symptoms). The patient’s cortisol levels, previously within normal range, increased to 30.75 µg/dL during the treatment period, representing a potentially significant neuroendocrine finding temporally associated with symptomatic improvement. However, the clinical significance of this observation remains uncertain, and no causal relationship to osteopathic treatment can be inferred. Continued endocrine and multidisciplinary follow-up remained clinically appropriate given the presence of coexisting pituitary and hormonal abnormalities. Although complete symptom resolution was not achieved, the patient experienced clinically significant improvement in balance perception, psychological distress, and overall functional capacity after her OCMM treatments. She also reported fewer falls and increased confidence ambulating without assistive devices. Notably, after years of refractory symptoms, the patient reported feeling relief immediately after undergoing OCMM treatment. At the one-year follow-up, serum cortisol levels were within normal range, though the temporal relationship to treatment remains unclear.

## Discussion

This report describes the case of a patient with PPPD refractory to conventional management, whose symptoms improved following identification and treatment of a cranial somatic dysfunction with OCMM. Despite extensive yet inconclusive evaluations by multiple specialists, a comprehensive osteopathic structural examination identified a superior sphenoid strain that had not been previously considered. Following two OCMM sessions, the patient experienced clinically meaningful improvement in balance perception, reduction in dizziness frequency, and significant improvement in anxiety and depressive symptoms. While causality cannot be established from a single case, these findings suggest that cranial somatic dysfunction may represent an underrecognized contributor to PPPD in select patients and that OCMM may serve as a useful adjunctive intervention when such dysfunctions are present.

PPPD is increasingly conceptualized as a functional vestibular disorder characterized by maladaptive interactions between vestibular processing, postural control, and emotional regulation. This framework aligns with current models emphasizing abnormal sensory reweighting and heightened threat perception, which may perpetuate symptom chronicity through feedback loops between vestibular dysfunction and anxiety [6]. Additionally, the overlap between PPPD and psychiatric comorbidities highlights the importance of integrative approaches that address both physiologic and psychological contributors to symptom persistence [2,3,5]. Neuroimaging and vestibular testing are often normal, contributing to diagnostic uncertainty and variable treatment responses. Dysregulation of autonomic tone and heightened threat perception are thought to perpetuate symptoms, creating a feedback loop in which anxiety exacerbates dizziness and dizziness reinforces anxiety. This bidirectional relationship aligns with emerging models implicating altered central vestibular processing, increased visual dependence, and persistent sympathetic arousal in PPPD [6]. In this context, interventions that influence autonomic balance and sensorimotor integration may have therapeutic relevance, even in the absence of structural lesions detectable on imaging.

From an osteopathic perspective, cranial base mechanics are particularly relevant to vestibular and autonomic function. Prior osteopathic literature has suggested that cranial somatic dysfunction may influence neurologic and autonomic processes through dural membrane tension and altered cranial base mechanics, providing a potential physiologic basis for symptom modulation [6,7]. OCMM is grounded in the primary respiratory mechanism, which describes the interrelationship between cranial bone mobility, dural membranes, cerebrospinal fluid fluctuation, and rhythmic motion palpated as the CRI [7,8]. Superior sphenoid strain is characterized by parallel motion of the sphenoid and occiput, resulting in vertical shearing forces that may contribute to symptoms such as dizziness, visual disturbance, and autonomic imbalance [9,10].

In osteopathic cranial concepts, dysfunction at the cranial base may influence symptoms beyond the head itself through tension transmitted along the dural membranes and altered cranial base mechanics [6,7,11]. Figure 5 illustrates the SBS, the cartilaginous articulation between the sphenoid and occiput at the cranial base, which serves as a key reference point for cranial strain patterns and dural membrane tension [7,11,12].

**Figure 5:**
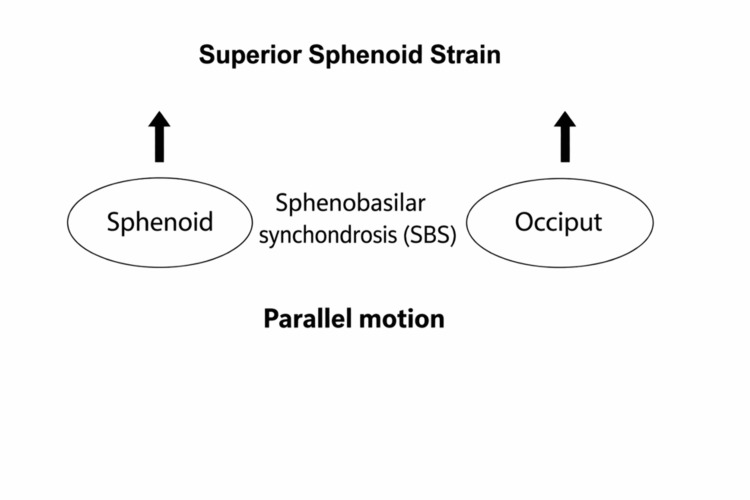
Schematic representation of superior sphenoid strain demonstrating parallel motion of the sphenoid and occiput at the sphenobasilar synchondrosis. Figure created using Microsoft PowerPoint.

Superior sphenoid strain, characterized by parallel motion of the sphenoid and occiput with vertical shearing, has been associated with symptoms such as dizziness, visual disturbance, headache, and emotional lability [7,9,12]. Altered tension within the dural membranes may influence cranial nerve function, including the vestibulocochlear nerve, as well as brainstem centers involved in balance and autonomic regulation [6,7,11]. These relationships are not visualized on routine neuroimaging but may be appreciated through skilled palpatory examination, underscoring a diagnostic domain unique to osteopathic practice [6,8,12].

OCMM may offer a mechanism for addressing these functional disturbances by restoring balanced motion at the SBS, reducing dural membrane strain, and supporting normalization of autonomic tone [6,7,11,12]. Techniques such as sphenobasilar decompression, balanced membranous tension, and compression of the fourth ventricle are traditionally understood to influence parasympathetic activity and cerebrospinal fluid dynamics [6,7,12]. In this patient, treatment directed at the identified superior sphenoid strain coincided with improvements in both vestibular symptoms and psychological distress. Although the patient had undergone extensive prior therapy without benefit, symptomatic improvement occurred temporally after OCMM, suggesting that cranial dysfunction may have been a contributing factor not previously addressed.

Notably, improvements were observed not only in balance perception but also in validated psychiatric screening measures, including normalization of the GAD-7 score and improvement in PHQ-9 severity. This finding supports a mind-body framework in which somatic interventions may influence psychological symptoms through shared neurophysiologic pathways [6,12]. While cortisol levels increased during the treatment period, this finding is interpreted cautiously. Cortisol is closely linked to anxiety, stress response, and hypothalamic-pituitary-adrenal axis activity, and the proximity of the sphenobasilar region to the sella turcica raises theoretical considerations regarding neuroendocrine regulation. However, cortisol measurements were not obtained immediately before and after treatment sessions, limiting interpretability, and no causal relationship can be inferred. Although the pituitary lesion identified on imaging was considered incidental by multiple specialists, its location within the sella turcica places it in close anatomic proximity to the sphenobasilar region, raising the possibility (without implying causality) that local sellar/suprasellar pathology could influence cranial base mechanics or dural membrane tension in select patients. The presence of coexisting structural and endocrine abnormalities in this patient required continued multidisciplinary evaluation, and the authors do not suggest that PPPD or psychiatric symptoms should be presumed to be exclusively functional in origin.

This case also illustrates how coexisting psychiatric diagnoses may influence clinical interpretation of physical symptoms [2,3,5]. A comprehensive osteopathic evaluation provided an alternative framework that incorporated structural, neurologic, and psychological factors without prioritizing one domain over another. The patient’s long-standing psychiatric diagnoses contributed to repeated dismissal of her physical symptoms, delayed exploration of alternative explanatory frameworks, and progressive functional decline [2,3,5]. By emphasizing palpatory diagnosis and whole-person assessment, osteopathic medicine may help mitigate diagnostic bias and restore clinical curiosity in patients whose symptoms have been prematurely attributed to psychiatric causes [3,4,5,6]. This approach has important implications for patient trust, engagement, and outcomes. Importantly, this patient’s presentation involved substantial functional impairment, recurrent falls, mobility limitations, psychiatric comorbidity, endocrine abnormalities, and structural findings, underscoring the multifactorial and medically complex nature of chronic vestibular symptom syndromes. Incorporating osteopathic training, particularly in comprehensive structural examination and whole-person assessment, may provide clinicians with additional diagnostic frameworks that can prevent diagnostic bias and improve patient-centered care.

Several limitations must be acknowledged. This is a single-patient case report and cannot establish causality or generalizability. Placebo effects, therapeutic context, and nonspecific clinician-patient interactions may have contributed to symptom improvement. Additionally, nonspecific neurophysiologic effects related to therapeutic touch and sensory modulation may also have contributed to the observed clinical changes. Outcomes relied heavily on patient-reported measures, and assessments were not blinded. Notably, palpatory assessment of the CRI and SBS has recognized limitations in inter-examiner reliability, and formal reproducibility testing was not performed in this case. Objective vestibular testing was not performed contemporaneously with treatment, and neuroendocrine measurements were limited. Comprehensive neuroendocrine and neuro-ophthalmologic evaluation, including formal pituitary functional testing and visual field assessment, was also not available for review, further limiting exclusion of alternative organic contributors to the patient’s symptoms. Future research should include controlled studies evaluating OCMM in PPPD, standardized cranial examination protocols, and objective outcome measures. Consideration should also be given to screening patients with chronic dizziness or psychiatric comorbidity for cranial somatic dysfunction as part of a comprehensive evaluation. Additional methodological limitations include the retrospective nature of the case review, incomplete availability of prior specialty evaluations and diagnostic studies, and other inherent limitations associated with single-patient case reports.

## Conclusions

This case highlights the potential clinical relevance of osteopathic cranial somatic dysfunction in patients with persistent postural-perceptual dizziness (PPPD), particularly when symptoms are refractory to conventional treatment in the setting of coexisting psychiatric conditions. In this patient, identification of a superior sphenoid strain through osteopathic structural examination and subsequent treatment with osteopathic cranial manipulative medicine (OCMM) were temporally associated with meaningful improvements in balance perception, dizziness frequency, and psychological distress. Although causality cannot be established, these findings suggest that cranial dysfunction may represent an underrecognized contributor to PPPD symptom persistence in select individuals. Importantly, this case shows the importance of comprehensive evaluation in patients with coexisting psychiatric comorbidities and demonstrates how an osteopathic whole-person approach may help mitigate bias by integrating structural, neurologic, and psychological assessment. The absence of definitive correlating findings on conventional neuroimaging does not necessarily exclude functional contributors to chronic vestibular symptoms that may be explored through osteopathic structural evaluation. In such cases, OCMM may offer a low-risk, patient-centered adjunctive intervention that complements existing vestibular, psychological, and pharmacologic therapies. While further investigation is needed, this case supports consideration of osteopathic structural and cranial evaluation in patients with chronic dizziness, and particularly those with coexisting anxiety or trauma-related disorders. Future research should focus on controlled studies examining the prevalence of cranial somatic dysfunction in PPPD, the reproducibility of cranial diagnostic findings, and the therapeutic role of OCMM in both functional vestibular disorders and psychiatric disorders. Incorporating osteopathic principles into the multidisciplinary care of patients with complex, overlapping somatic and psychological symptoms may provide an additional framework for comprehensive assessment while helping reduce premature diagnostic closure.
